# The Practice of Surgery

**Published:** 1911-01

**Authors:** 


**The Practice of Surgery. By James Gregory Mumford,** M.D., Visiting Surgeon to the Massachusetts General Hospital; Instructor in Surgery in the Harvard Medical School. Octavo, pp. 1015. With 682 illustrations. Philadelphia and London: W. B. Saunders Co. 1910. (Cloth, $7.00.)

The accuracy of diagnosis, and the technic of surgical practice has developed, broadened, and increased greatly during the years just past, more so perhaps than in any period of our history. Numerous, indeed, are the textbooks that have been published on the subject; the current that feeds the mill of "know how" is never still.

The author belongs to the younger generation of sugeons, but he is a man whose experience and standing as an author, a teacher, and a surgeon, fit him well for the work he has done.

Mumford's book does not assume to deal comprehensively with the entire subject of surgery. This cannot be done in one volume of moderate size, hence the author has omitted consideration of principles, but confines himself rather to a treatise that teaches practical surgery, the surgery that every general practitioner will see at the bedside or in his consulting room, and the kind that he wants to know about. The plan of the book is out of the usual method, inasmuch as it takes up surgical diseases in their order of interest, importance, and frequency, so far as it may be done with due regard to proper sequence.

The work is divided into six parts of thirty-one chapters. The first part of the book is taken up with discussion of diseases of the abdomen, followed by diseases of the female organs of generation, genitourinary organs, diseases of the chest, face, and neck, the head and spine, in the order given. The last part of the book, 274 pages, is given over to minor surgery, every line of which teaches something This work will be cordially welcomed by the profession. J. A. R.

